# Modeling mtDNA hypermethylation vicious circle mediating Aβ-induced endothelial damage memory in HCMEC/D3 cell

**DOI:** 10.18632/aging.103699

**Published:** 2020-09-28

**Authors:** HaoChen Liu, Hong Zhang, Yixuan Zhang, Sheng Xu, Huimin Zhao, Hua He, XiaoQuan Liu

**Affiliations:** 1 Center of Drug Metabolism and Pharmacokinetics, China Pharmaceutical University, Nanjing 210009, China

**Keywords:** Alzheimer’s disease, endothelial cells damage memory, mtDNA hypermethylation, vicious circle, α-oxoglutarate

## Abstract

It is well accepted that accumulation of beta-amyloid (Aβ) may involve in endothelial dysfunction during the Alzheimer’s disease (AD) progression. However, cerebrovascular function cannot be improved by removing Aβ in AD animal models. The reasons for these paradoxical results still remain to be further investigated. We hypothesize that Aβ exposure may cause persistence damage to cerebral endothelial cell even after Aβ is removed (termed as cerebrovascular endothelial damage memory) mitochondria DNA (mtDNA) hypermethylation is assumed to be involved in this process. The aim of this study is to investigate whether Aβ exposure induces cerebrovascular endothelial damage memory in endothelial cells and mtDNA hypermethylation involves in this process. The hCMEC/D3 cell is treated with *Aβ*_1–42_ for 12h and then withdraw *Aβ*_1–42_ for another 12h incubation to investigate whether cerebrovascular endothelial damage memory exists in endothelial cells. The levels of mtDNA methylation and cell vitality were not improved by removing *Aβ*_1–42_ after 12h *Aβ*_1–42_ incubation which suggested that the cerebrovascular endothelial damage memory may exist in endothelial cells. Kinetics model analysis suggested that mtDNA hypermethylation involves in initiating the cerebrovascular endothelial damage memory otherwise α-oxoglutarate (AKG) exhaustion plays a vital role in maintaining this process. DNA methylation inhibitor decitabine and AKG supplement may relieve the cerebrovascular endothelial damage memory dose dependently. This study provides a novel feature of cerebrovascular endothelial damage induced by Aβ.

## INTRODUCTION

Alzheimer’s disease (AD) is the leading cause for dementia which has been considered as one of the major public health problem worldwide [1]. The amyloid cascade hypothesis suggested that amyloid β (Aβ) accumulation is a major pathology hallmark in the development of AD [2, 3]. Therefore, according to the hypothesis, multiple anti-bodies targeted at Aβ (e.g. Solanezumab, Bapineuzumab and Crenezumab, etc) are tested in AD patients [4]. Unfortunately, none of these anti-bodies exhibit efficacy in clinical trails [5].

Previous researches suggested that the anti-bodies targeted at Aβ failing in clinical trials may be due to that they are not given in the early stage of AD [5, 6]. However, other research proposed a different reason for anti-Aβ immunotherapies lacking efficacy. It is widely accepted that cerebrovascular system may play an important role in Aβ clearance [7]. Therefore, Qi et al suggested that removing Aβ invalid at improving cerebrovascular damage may be related to the anti-Aβ immunotherapies lacking efficacy [8]. Previous reports could provide more evidences for this hypothesis. When the AD animal models are treated with anti-Aβ antibodies, they are effective at removing the Aβ plaques, but void at preventing hemorrhages which may be related to the cerebrovascular damage [9–12]. In other words, Aβ may impair the cerebrovascular function, but the cerebrovascular function can not be improved by removing Aβ [10]. At present there is still no reasonable explanation for above paradoxical results. Therefore, it is necessary to investigate the reasons for lacking efficacy of removing Aβ on cerebrovascular function improvement.

The researches on the diabetes “metabolic memory” phenomenon may provide enlightenments for the investigation on the persistent endothelial dysfunction of AD. Metabolic memory phenomenon defined as the persistence of diabetes complications even after glycemic control has been pharmacologically achieved [13]. Especially, the Metabolic memory phenomenon is associated to the endothelial dysfunction [14]. There is similar phenomenon observed in AD animals that removing Aβ can not improve endothelial dysfunction. Therefore, we assume that the damage memory phenomenon may exist in Aβ induced cerebrovascular endothelial cell damage.

**Figure 1:**
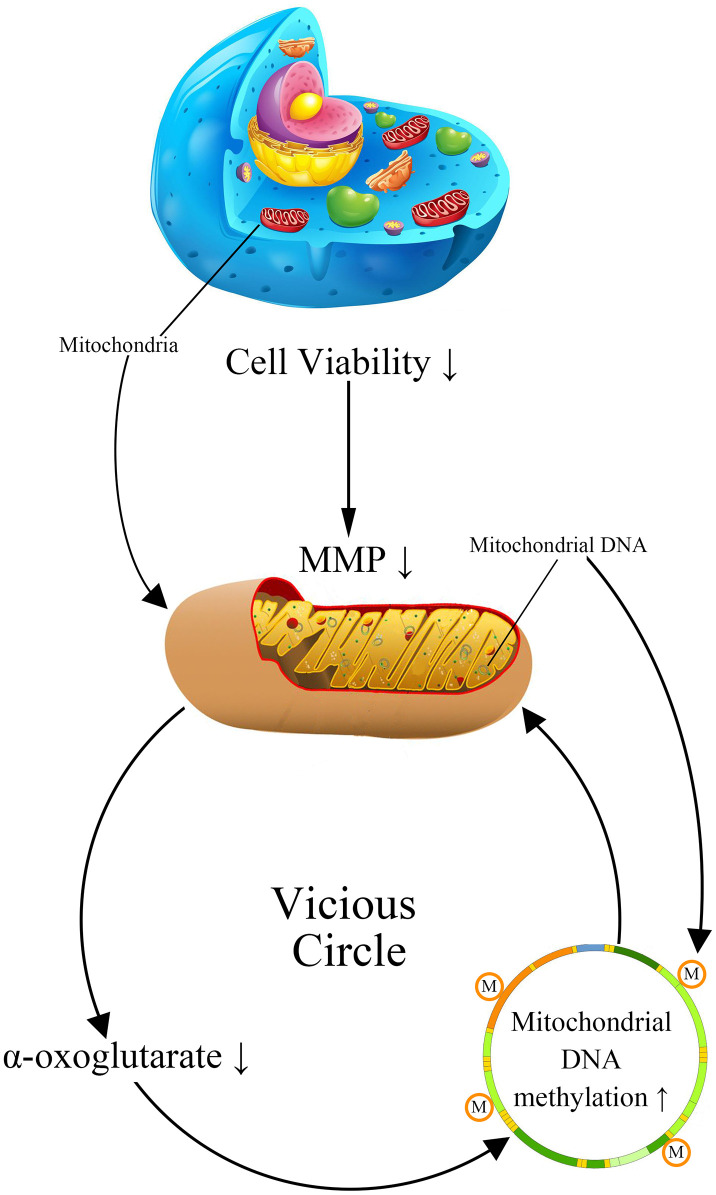
**The cerebrovascular endothelial cell damage memory vicious circle.**

Previous researches have suggested that mitochondria DNA (mtDNA) damage plays an important role in metabolic memory [15, 16]. As the damage of mtDNA is observed in AD patients, it is rational to suppose that mtDNA may involve in the formation of cerebrovascular endothelial damage memory [17–19]. The mtDNA damage in AD patients includes multiple types: mutations, deletion, insertion, and hypermethylation [20, 21]. The confirmation of both 5-methylcytosine (5mC) and 5-hydroxymethyl cytosine (5-hmC) occurring in mtDNA prompted a resurgence of interest in the roles of mtDNA methylation in AD [22]. The global mtDNA methylation level is about 0.3–0.5% of total cytosine residues in which the regulatory D-loop region represents one of the most methylated sites [23]. The variation of mtDNA methylation level is observed in AD animal models, AD patients postmortem brains and blood, which is suggested to be related to the mitochondria dysfunction [20, 24, 25]. Therefore, we assume that the variation of mtDNA methylation level may involve in the formation of cerebrovascular endothelial damage memory. The formation of cerebrovascular endothelial damage memory may be related to the vicious circle mediated by mtDNA hypermethylation. We assumed that vicious circle may be initialized by the increased mtDNA methylation level which may collapse the MMP, then the dysfunction of mitochondria may reduce the production of AKG which is essential for mtDNA demethylation; AKG exhaustion may exacerbate mtDNA hypermethylation resulting in a vicious circle of endothelial cell damage (Figure 1).

The aim of this study is to investigate that whether the damage memory process may exist in cerebrovascular endothelial cell and the role of mtDNA methylation in this process. Additionally, a mechanism based kinetic progression model is developed to investigate the dynamic process of cerebrovascular endothelial cell damage as well as the method for improving the cerebrovascular endothelial cell damage memory. The kinetics model could provide three aspects information. Firstly, the kinetics model provides prediction for the formation time of damage memory. Secondly, the kinetics model provides clues for the key player during the formation of damage memory. Thirdly, the kinetics provides potential strategies for improving the damage memory. Our research provides new insight into the AD cerebrovascular endothelial cell dysfunction and the new idea for the improvement of cerebrovascular endothelial function.

## RESULTS

### Withdrawing Aβ does not improve hCMEC/D3 cell vitality

**Figure 2:**
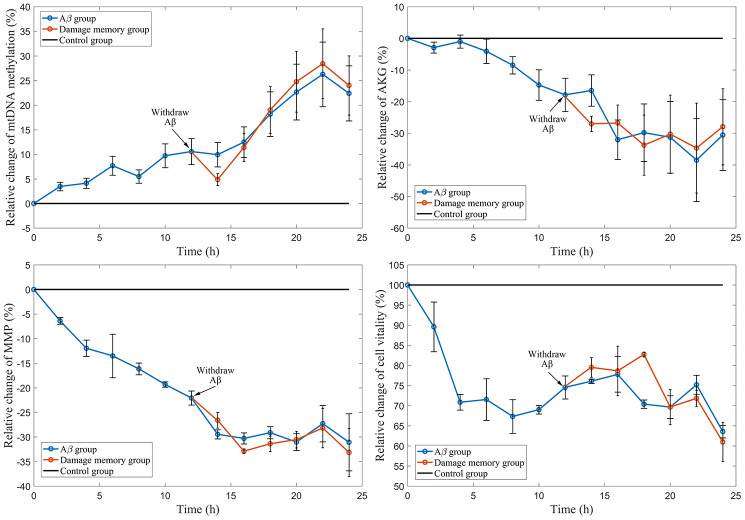
**The time course of relative change of mtDNA methylation, AKG, MMP, and cell vitality compared to the control group.** The black line represent the control group level which is normalized to 100%. The blue line represent Aβ group level. The red line represent the damage memory group.

The results (Figure 2D) show that the cell vitality in Aβ group decreases during *Aβ*_1–42_ incubation. The cell vitality in the damage memory group can not be increased by removing *Aβ*_1–42_ which has no significant difference (P>0.05) compared with Aβ group. These results suggested that the damage memory may exist in endothelial cell. In other words, if endothelial cell exposes to *Aβ*_1–42_ for a certain time, the damage induced by *Aβ*_1–42_ may not be improved by withdrawing *Aβ*_1–42_. Furthermore, Our result (Figure 2A–2C) suggested that the mtDNA methylation level increased during *Aβ*_1–42_ incubation but it did not decrease after *Aβ*_1–42_ is withdrawn meanwhile the the levels of α-oxoglutarate (AKG) and mitochondrial membrane potential (MMP) was reduced during *Aβ*_1–42_ incubation nevertheless they did not recover after *Aβ*_1–42_ was withdrawn. Therefore, our results suggested that the damage memory may exist in hCMEC/D3 cell.

### Inhibiting mtDNA methylation relieves the endothelial damage memory

**Figure 3:**
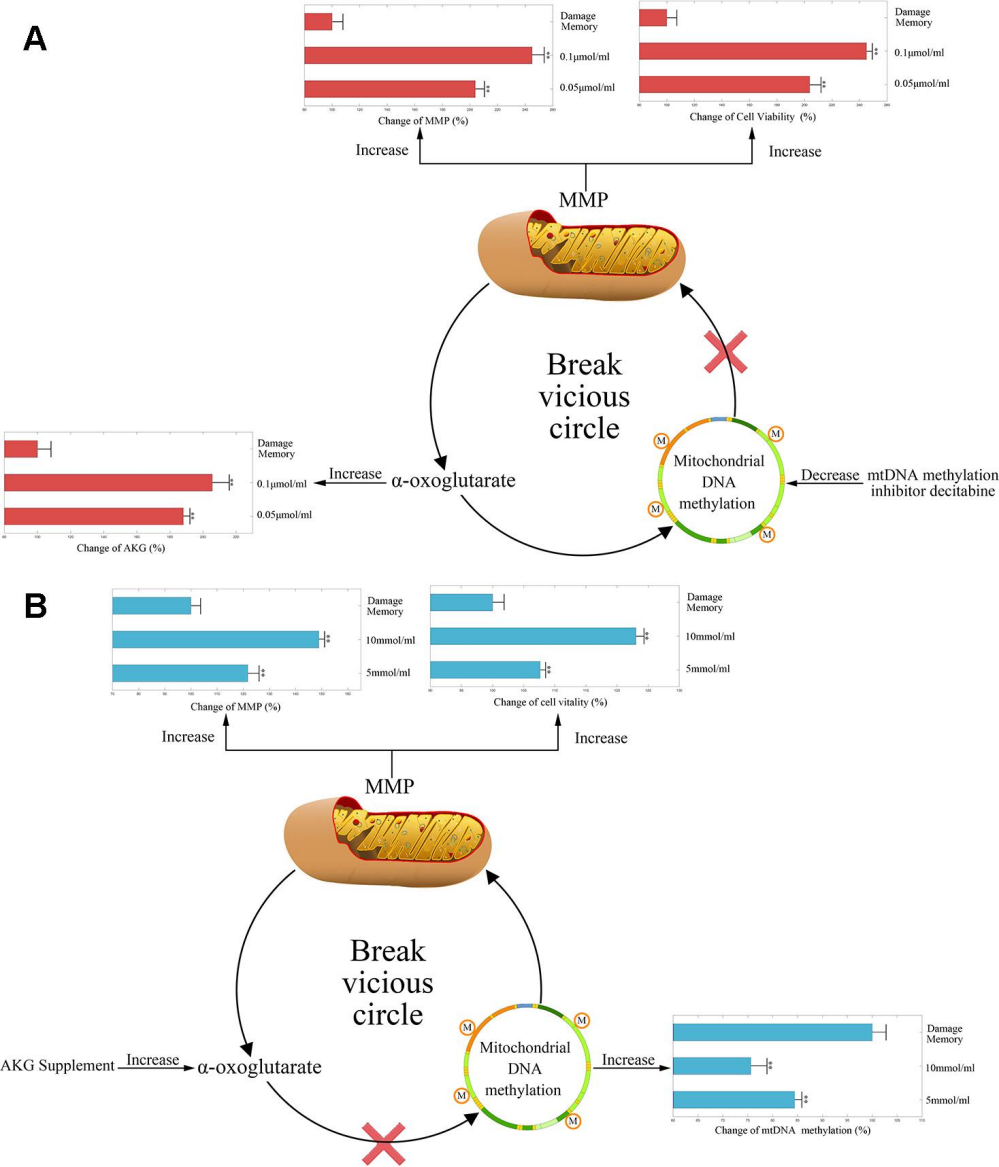
****(**A**) The change of AKG, MMP, and cell vitality in decitabine treated hCMEC/D3 cell. The control group data is normalized to 100%. (**B**) The change of mtDNA methylation, MMP, and cell vitality in AKG treated hCMEC/D3 cell. The control group data is normalized to 100%. ** p<0.01 * p<0.05.

During the endothelial damage memory formation process, the level of mtDNA methylation elevated continuously. Therefore, we wonder that whether demethylation of mtDNA may relieve endothelial damage memory. There are two ways to demethylate mtDNA. Firstly, DNA methyltransferase inhibitor decitabine may reduce the mtDNA methylation by inhibiting the activity of DNA methyltransferase (DNMT). Secondly, as AKG is a vital cofactor of ten eleven translocation protein (TET) which is the demethylation enzyme, AKG supplement also may stimulate the demethylation of mtDNA. Therefore, both methods are used to test whether the mtDNA methylation level may affect the formation of the endothelial damage memory. In the first experiment, decitabine is used to inhibit the methylation of mtDNA. The results are shown in Figure 3A. Compared with the damage memory group, the levels of AKG, MMP and cell vitality in decitabine treated groups increased significantly (p<0.05). The variations of above biomarkers levels are dose dependent. Therefore, our reults suggested that inhibiting mtDNA methylation relieve the endothelial damage memory.

In the second experiment, AKG supplement is used to increase the activity of TET. The results are represented in Figure 3B. Compared with memory group, the levels of MMP and cell vitality in AKG treated groups increased significantly (p<0.05) meanwhile the level of mtDNA methylation decreased significantly (p<0.05). The variations of above biomarkers levels are dose dependent. Therefore, our reults suggested that AKG supplement relieves the endothelial damage memory.

### mtDNA methylation and AKG play different roles in the dynamic process of endothelial damage memory

**Figure 4:**
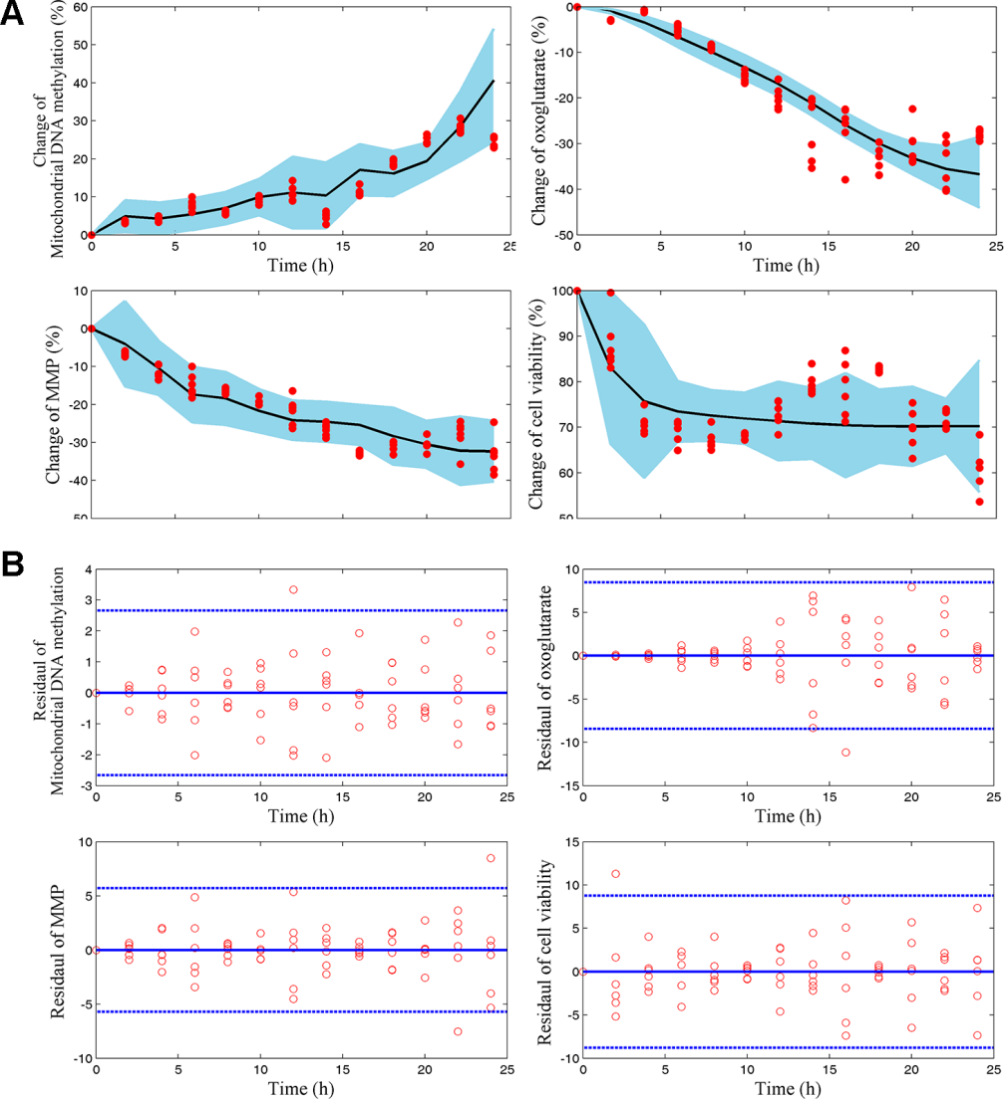
****(**A**) Visual predicted check (VPC) for mtDNA methylation, AKG, MMP, and cell vitality. The shaded area represents the 95% confidence interval of the simulated median value. The line represents the median value of observed value. The red scatters represent observed values. (**B**) Scatter plots of predicted residuals vs. time for mtDNA methylation, AKG, MMP, and cell vitality.

We wonder that whether mtDNA methylation and AKG may play different roles in the endothelial damage memory kinetic process. To test this hypothesis, a mechanism based kinetic progression model is developed. The visual predictive check (VPC) for this model is represented in Figure 4A, 4B. The VPC plots show that the observed average data falls within 95% prediction confidence interval. The bootstrapping values of estimated model parameters (Table 1) remain near the final parameters estimation with relative low coefficient of variances (CV). Therefore, the goodness of fit for the mechanism based kinetic progression model is satisfactory.

**Table 1:** Mechanism based kinetic process model parameters and bootstrap validation.

**Parameters**	**Estimate**	**CV(%)**	**CI**		**Bootstrap**
			LL	UL	
**$k_{in}^{MMP}$**	0.02	3.53	0.10	0.22	0.16
**$k_{out}^{MMP}$**	0.80	0.95	0.04	1.57	0.80
**$E_{\max}^{Me-mtDNA}$**	0.60	0.54	0.28	0.92	0.60
**$EC_{50}^{Me-mtDNA}$**	0.03	0.72	0.01	0.06	0.03
**$k_{in}^{AKG}$**	0.17	0.37	0.11	0.24	0.18
**$k_{out}^{AKG}$**	0.12	0.70	0.04	0.20	0.13
**$k_{in}^{Me-mtDNA}$**	-0.02	-0.92	-0.03	0.00	-0.02
**$k_{out}^{Me-mtDNA}$**	0.04	0.70	0.01	0.07	0.04
**$E_{\max}^{AKG}$**	5.53	0.40	3.31	7.75	5.53
**$EC_{50}^{AKG}$**	0.20	0.96	0.01	0.38	0.20
**$E_{{}_{}^{A\beta }}$**	0.61	0.45	0.34	0.88	0.61
**$k_{in}^{MTT}$**	0.94	0.84	0.15	1.72	0.94
**$k_{out}^{MTT}$**	0.26	0.16	0.21	0.30	0.26
**$E_{\max}^{MMP}$**	0.04	0.79	0.01	0.08	0.04
**$EC_{50}^{MMP}$**	1.04	0.79	0.22	1.85	1.04

**Figure 5:**
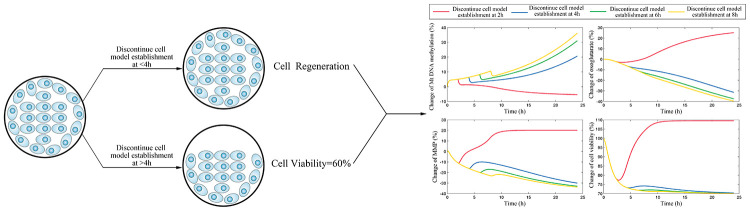
**The simulation results for scenario I which investigate the time of cerebrovascular endothelial cell damage memory formation.**

**Figure 6:**
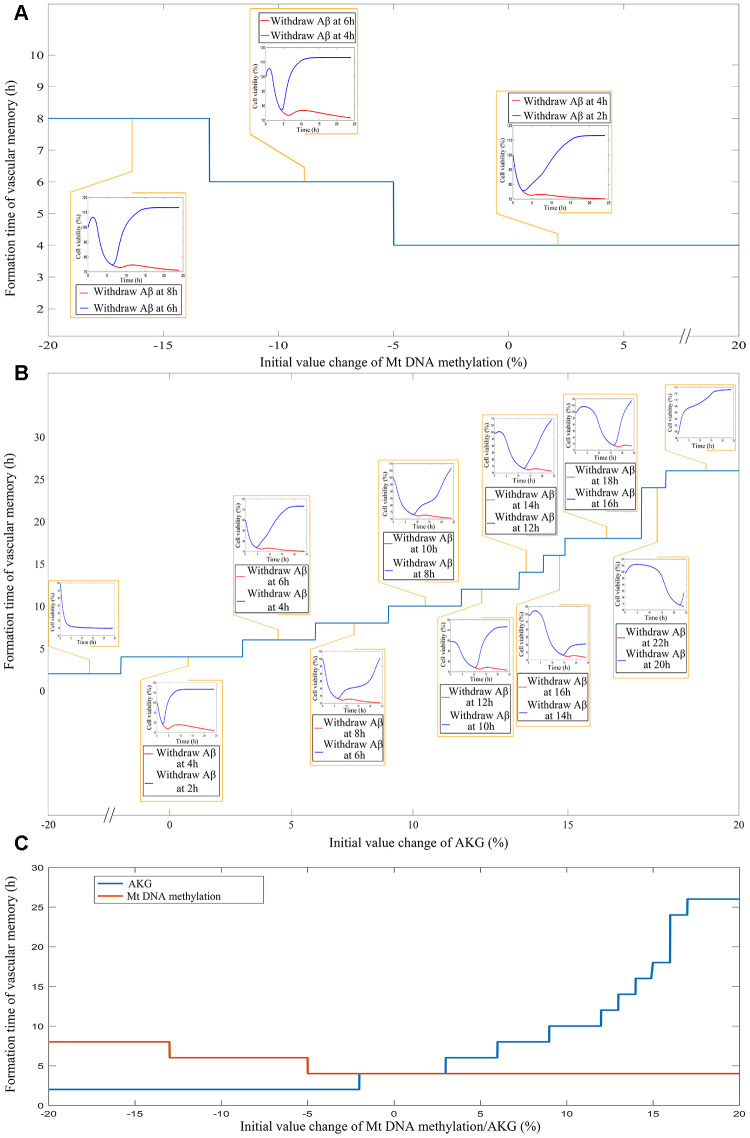
****(**A**) The impact of different levels of mtDNA methylation on the cerebrovascular endothelial cell damage memory formation time. (**B**) The impact of different levels of AKG on the cerebrovascular endothelial cell damage memory formation time. (**C**) The summary plot of the impact of different levels of mtDNA methylation and AKG on the cerebrovascular endothelial cell damage memory formation time.

**Figure 7:**
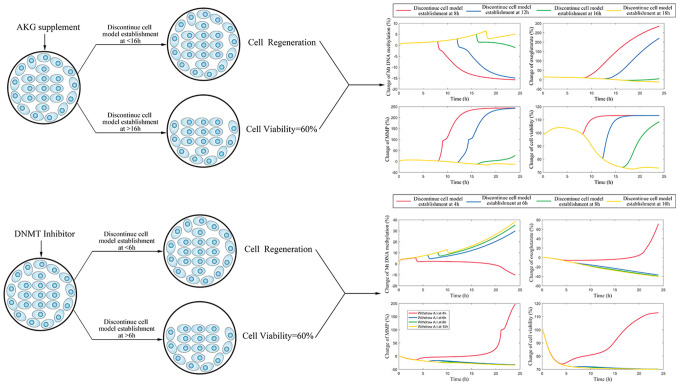
****(**A**) The simulation for change of mtDNA methylation, AKG, MMP, and cell vitality in AKG supplement treated cells. (**B**) The simulation for change of mtDNA methylation, AKG, MMP, and cell vitality in DNMT inhibitor treated cells.

After the internal validation of the mechanism based kinetic progression is performed, simulations based on this model are conducted. The simulations are performed based on three scenario. The aim for the first scenario is to investigate the time of endothelial damage memory formation. The relevance of this simulation is to provide a baseline data for comparing the effects of different levels of mtDNA methylation and AKG on the time of endothelial damage memory formation. The results of the first simulation are shown in the Figure 5. The results suggested that when the cell is treated with *Aβ*_1–42_ for more than 4h, the levels of mtDNA methylation, AKG, MMP and cell vitality may not be recovered by withdrawing *Aβ*_1–42_. In other words, the baseline of endothelial damage memory formation time might be 4h post *Aβ*_1–42_ treatment in hCMEC/D3 cell. After the base line time of endothelial damage memory formation is estimated, the simulation of the second scenario is performed. In this scenario, the level of mtDNA methylation or AKG is changed and then the time of endothelial damage memory formation is estimated. The results of above simulation are shown in Figure 6A–6C. Changing the level of mtDNA methylation or AKG may alter the time of endothelial damage memory formation. Particularly the variation of the endothelial damage memory formation time is more sensitive to changing of the AKG level than that of the mtDNA methylation level. In the third scenario, the methods for relieving the endothelial damage memory were investigated. The effects of decitabine or AKG supplement on relieving the endothelial damage memory were estimated. The results of the above simulation are shown in Figure 7A, 7B. When the cells are treated with decitabine, the time of endothelial damage memory formation is delayed to 6h post *Aβ*_1–42_ incubation. When the cells are treated with AKG supplement, the time of endothelial damage memory formation is delayed to 18h post *Aβ*_1–42_ incubation. These results suggested that AKG supplement may be a potential method for delaying the formation of endothelial damage memory.

### Different roles of decitabine and AKG supplement in delaying the formation of endothelial damage memory

**Figure 8:**
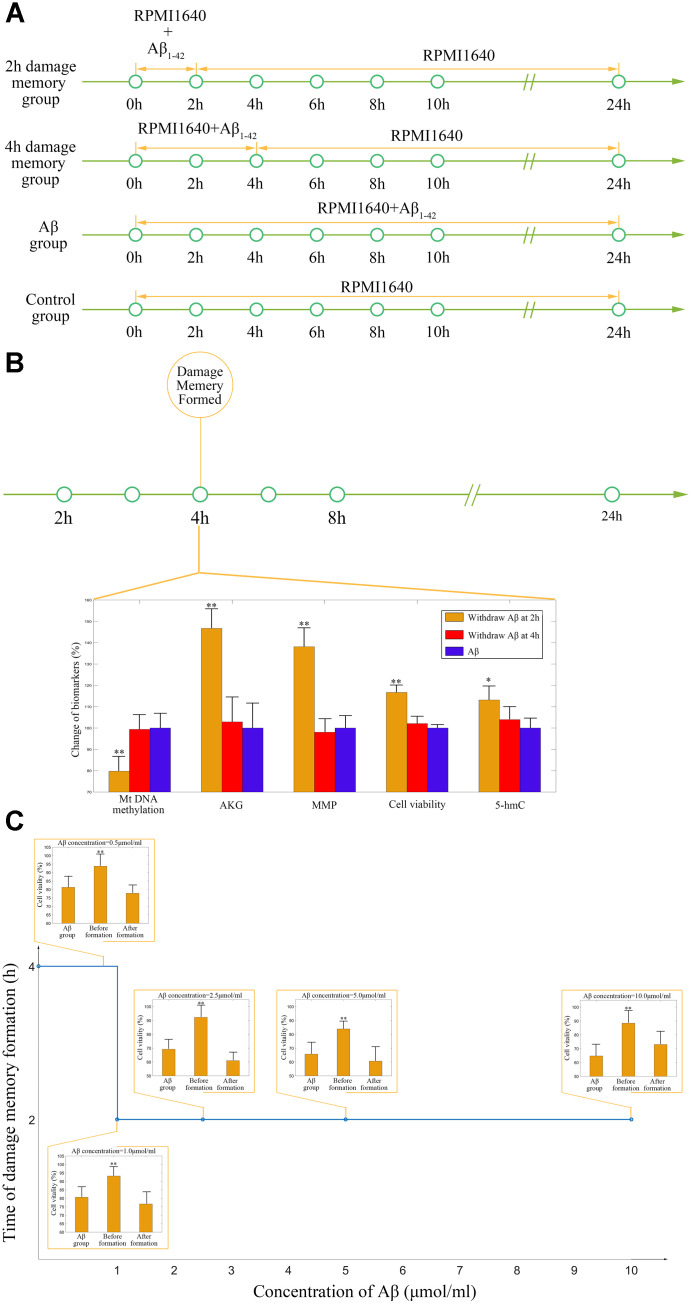
****(**A**) Experimental protocol for determining time of cerebrovascular endothelial cell damage memory formation. (**B**) The experimental validation of cerebrovascular endothelial cell damage memory formation time. (**C**) The damage memory formation time with different concentration of Aβ incubation.

As the above simulations suggested that the AKG supplement may delay the formation of endothelial damage memory, cell experiments are performed to test this hypothesis. To test that whether AKG supplement can delay the formation of endothelial damage memory, the baseline time of endothelial damage memory formation should be determined. The above simulation suggested that the baseline time for endothelial damage memory formation may be 4h after *Aβ*_1–42_ incubation. Therefore, the cell experimets are designed according to the simulation (Figure 8A). The cell vitality in 2h memory group recovered whereas it did not relive in 4h memory group after Aβ is withdrawn (Figure 8B). Compared with Aβ group, the cell vitality in 2h memory group had significant difference (p<0.05) but it had no significant difference in 4h memory group (Figure 8B). The results suggested that the baseline time of endothelial damage memory formation might be 4h after *Aβ*_1–42_ incubation. Compared with Aβ group, the level of mtDNA methylation in 2h memory group decreased significantly (p<0.01) while the levels of AKG and 5-hmC increased significantly (p<0.05). In addition, to investigate the effect of different concentration of Aβ on the time of damage memory formation, the formation time of the damage memory were estimated when the cells were incubated with different concentrations of Aβ. The results suggested that decreased Aβ_1–42_ concentration may delay the formation time of the damage memory (Figure 8C).

**Figure 9:**
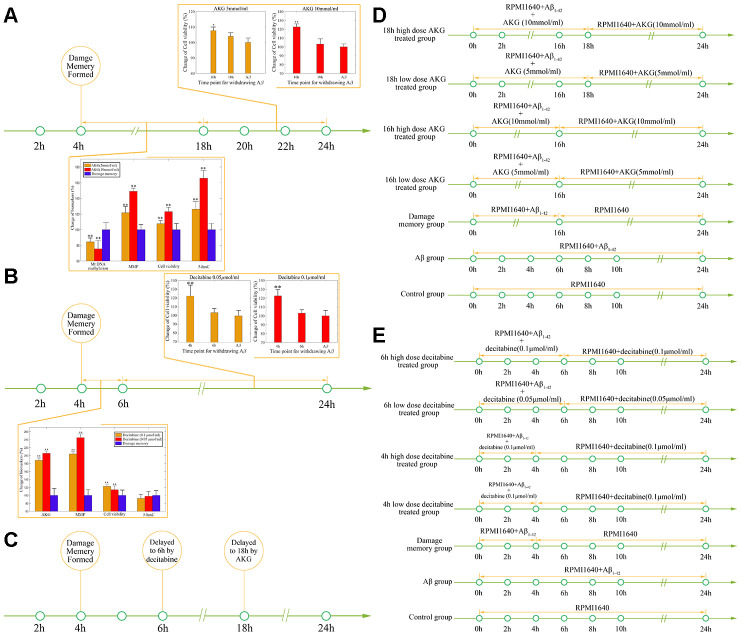
****(**A**) The effect of AKG supplement on delaying the formation of cerebrovascular endothelial cell damage memory formation. (**B**) The effect of decitabine on delaying the formation of cerebrovascular endothelial cell damage memory formation. (**C**) Summary of the effect of decitabine and AKG supplement on delaying the formation of cerebrovascular endothelial cell damage memory formation. (**D**) Experimental protocol for evaluating the effect of AKG supplement on delaying the formation of cerebrovascular endothelial cell damage memory. (**E**) Experimental protocol for evaluating the effect of decitabine on delaying the formation of cerebrovascular endothelial cell damage memory.

After the baseline time of endothelial damage memory formation is estimated, the effect of decitabine or AKG supplement on delaying endothelial damage memory formation is investigated. The above simulation shows that the formation of endothelial damage memory may be delayed to 6h or 18h after *Aβ*_1–42_ incubation by decitabine or AKG supplement respectively. Therefore, the cell experimets were desinged according to the simulation (Figure 9D–9E). The results of AKG supplement group are shown in Figure 9A. The results suggested that the cell vitality in 16h AKG groups (including low dose and high dose) is significantly higher (p<0.05) than that in 18h AKG (including low dose and high dose) groups and damage momery group meanwhile there is no significant difference between 18h AKG (including low dose and high dose) groups and damage momery group. Compared with the damage momery group, the levels of MMP, AKG, 5-hmC, and cell vitality increased significantly (p<0.05) in AKG treated cells. Therefore, treating with AKG may delay the endothelial damage memory formation to 18h after *Aβ*_1–42_ incubation.

The results of decitabine treatment are shown in Figure 9B. The cell vitality in 4h decitabine group (including low dose and high dose) is significantly higher (p<0.05) than that in 6h decitabine (including low dose and high dose) group and damage momery group meanwhile there is no significant (p>0.05) difference between 6h decitabine (including low dose and high dose) group and damage momery group. Compared with the damage momery group, the levels of MMP and cell vitality increased significantly (p<0.05) in decitabine treated cells. Therefore, treating with decitabine may delay the endothelial damage memory formation to 6h after *Aβ*_1–42_ incubation. Therefore, the experiments results suggested that compared with decitabine, AKG supplement may exhibit better effects on delaying the formation of endothelial damage memory (Figure 9C).

## DISCUSSION

The cerebrovascular endothelial cell dysfunction induced by Aβ is an important feature of AD [10, 26]. Despite the mitochondrial dysfunction is involved in cerebrovascular endothelial cell dysfunction, the role of mtDNA modifications has been largely ignored in this damage process. In this study, the role of mtDNA methylation in cerebrovascular endothelial cell dysfunction termed as endothelial cell damage memory is illustrated. The appearances of irreversible damage and damage memory may be similar. But their concepts are different. The irreversible damage is believed to be impossible to be improved. For example, the nerve damage and cerebral atrophy observed in AD patients are irreversible damages which are believed impossible to be cured at present. The endothelial damage memory induced by Aβ is a phenomenon that the cerebral vascular endothelial may memory the Aβ existence even after Aβ has been removed. The damage induced by Aβ exposure may still exist after Aβ is removed. The most significant difference between irreversible damage and damage memory is that the irreversible damage is impossible to be improved whereas the damage memory may not be relieve by removing Aβ but it can be improved by other methods such as AKG supplement.

mtDNA is a circular, double-stranded DNA molecule which comprises 37 genes and 13 of those encode for polypeptides required for the electron transport chain (ETC) [27, 28]. The modification of mtDNA involves in the addition of a methyl group on the cytosine base giving rise to 5mC [28]. Previous research had linked the presence of mtDNA lesions and the loss of MMP [29]. Meanwhile the hypermethylation of mtDNA may be one of the sources of mtDNA lesions [15]. Therefore, we wonder that whether the variation of global mtDNA methylation may affect the mitochondria function. Our study suggested that the global hypermethylation of mtDNA is observed in the Aβ induced endothelial cells which might be related to the collapse of MMP. As previous research had suggested that DNA methyltransferase (DNMT) decreased significantly in AD patients’ brains, we assumed that the hypermethylation of mtDNA may be due to the dysfunction of demethylation process [30]. mtDNA may be demethylated by TET whose cofactor is AKG [31]. The decreased AKG may prevent mtDNA demethylation. Therefore, we assumed that Aβ may increase the mtDNA methylation level which may collapse the MMP, then the dysfunction of mitochondria may reduce the production of AKG; meanwhile, as AKG is essential for mtDNA demethylation, low level of AKG may exacerbate mtDNA hypermethylation resulting in a vicious circle of endothelial cell damage.

Furthermore, the dynamic process of cerebrovascular endothelial cell damage memory formation is investigated by the mechanism based kinetic progression model. According to our model, the progression of cerebrovascular endothelial cell damage memory might be divided into two phases. The first phase termed as the formation phase is defined that the cell vitality can be recovered by removing *Aβ*_1–42_. The second phase termed as the maintenance phase is defined that the cell vitality can not be recovered by removing *Aβ*_1–42_. mtDNA global methylation and AKG may play different roles in different phases. The increased mtDNA global methylation may cause mitochondria dysfunction which initializes the damage vicious circle. Once the vicious circle forms, the cell vitality is not sensitive to the variation of mtDNA global methylation. But in the maintenance phase, the cell vitality is sensitive to the change of AKG level. Therefore, it is suggested that AKG may play an important role in maintaining the vicious circle. In summary, the increased mtDNA global methylation initialized the vicious circle and the AKG exhaustion plays a vital role in the maintenance of the vicious circle.

According to the kinetic characters of the vicious circle, two strategies, inhibiting mtDNA methylation and AKG supplement, for improving the endothelial function are investigated. Compared with inhibiting mtDNA methylation, AKG supplement may exhibit better effect on delaying the damage memory formation. The reason for this hypothesis may lie on the different roles of DNMT and TET. Previous clinical researches have suggested that DNMT level decreases and TET level increases in AD patients [30, 32, 33]. The variation of DNMT and TET seems to be conducive to the demethylation of mtDNA. However, the hypermethylation of mtDNA is observed which suggested that the variation of DNMT and TET may not play dominant roles in the damage memory formation. The increased mtDNA methylation level may be due to the demethylation dysfunction caused by exhaustion of the TET critical cofactor AKG. As DNMT may not play a dominant role in the damage memory formation, inhibiting DNMT may not exhibit potent effect on delaying the damage memory formation. Meanwhile AKG exhaustion may play an important role in the damage memory formation. Therefore, compared with inhibiting DNMT, AKG supplement may have better effect on delaying the damage memory formation.

In this study, the kinetic progression of cerebrovascular endothelial cell damage memory vicious circle is demonstrated. mtDNA global hypermethylation involves in the initialization of the vicious circle. AKG exhaustion plays an important role in the maintenance of the vicious circle. Meanwhile AKG supplement may be a potential method for improving the cerebrovascular endothelial cell damage memory. The present study provides a new insight into cerebrovascular endothelial damage in AD progression.

## MATERIALS AND METHODS

### Research framework

**Figure 10:**
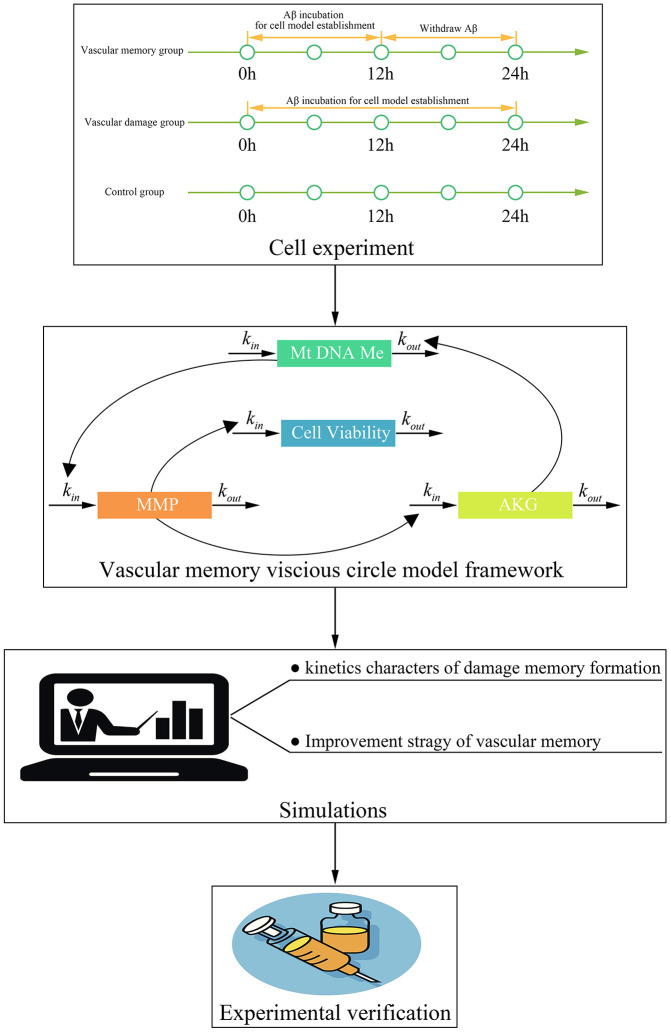
**The framework of this study.**

This study contains four steps (Figure 10). Firstly, cell experiments are performed to investigate that whether the damage memory exists in endothelial cells and obtain the data for the kinetics process of cerebrovascular endothelial cell damage. Secondly, a mathematical model is developed to describe the above kinetics process. Thirdly, simulations based on the above model are performed to investigate the kinetic characters of the damage process and improvement method of cerebrovascular endothelial cell damage. Fourthly, the improvement method proposed by the above simulations are validated by cell experiments.

### Cell culture

hCMEC/D3 cells were cultured in complete RPMI 1640 and seeded on glass coverslips in 6-well plates for mtDNA methylation analysis, 24-well plates for HPLC and MMP assays, or 96-well plates for cell vitality assays. All cell lines were maintained at 37°C and 5% CO_2_. Cell lines were validated by short tandem repeat (STR) profiling.

### Cell treatment

Aβ peptide is used to prepare AD cerebrovascular endothelial cell dysfunction model. The stock solution of Aβ peptide (100μM) is prepared by dissolving 1mg freeze-dried Aβ peptide powder in 2208μL PBS and 45μL DMSO. The stock solution is diluted to 2.5μM with complete RPMI 1640 solution for in vitro model preparation. To investigate that whether the cerebrovascular endothelial cell dysfunction memory exists in endothelial cell, the hCMEC/D3 cells are divided into three groups (Figure 10A). For the first group (control group), the hCMEC/D3 cells were cultured in complete RPMI 1640. For the second group (the Aβ group), the hCMEC/D3 cells are incubated with complete RPMI 1640 containing 2.5μM Aβ peptide for 24h. For the third group (the damage memory group), the hCMEC/D3 cells are incubated with 2.5μM Aβ peptide for 12h and then withdraw Aβ for anther 12h incubation. For all three groups, the cell samples are collected at 0h, 2h, 4h, 6h, 8h, 10h, 12h, 14h, 16h, 18h, 20h, 22h, 24h for mtDNA methylation, AKG, mitochondrial membrane potential (MMP) and cell vitality measurement. For mechanistic experiments, the cells in the damage memory group are treated with decitabine (a DNA methyltransferases inhibitor) and AKG [34].

### mtDNA methylation analysis

In this study, the global mtDNA methylation level is determined by LC-MS/MS. Firstly, the mtDNA is extracted by the protocol reported in a previous research with commerce plasmid miniprep kit [35]. To avoid the contamination of genomic DNA, we did not use the traditional DNA extraction method phenol–chloroform extraction. Instead, DNA ion exchange columns are applied to extract mtDNA in this study. The specific length of DNA fragments can bind to ion exchange columns and other components such as small molecules, DNA fragments in other length, proteins, etc. can not be retained by the column [36, 37]. The DNA ion exchange columns which can retain about 1kbp to 9kbp DNA fragments is selected. As the genomic DNA fragments are much longer than mtDNA. The genomic DNA fragments can not bind to the ion exchange column whereas the mtDNA can be retained by the column. Hence, the mtDNA is separated from genomic DNA. In this study, a ion exchange columns based commercial kit is used to extract mtDNA. The mtDNA extract (containing about 1 μg DNA) was acid-hydrolyzed by the following protocol [38]. 50μL of mtDNA extract was dried by nitrogen at 37°C. The residue was mixed with 100μL of 88% aqueous formic acid and then hydrolyzed at 130°C for 30 min. After the rest of formic acid was evaporated under nitrogen at 37°C, 5μL IS solution (500ng/ml acyclovir in water) and 50μL methanol was added to the residue. Then the mixture was centrifuged for 10 min at 15000 rpm under 4°C. 30μL of the supernatant was extracted for analysis by LC-MS/MS.

The prepared samples were injected into a Lichrospher NH_2_ column (5.0μm, 2.1×150mm). The mobile phase consisted of acetonitrile (phase A) and water containing 20mmol/L ammonium bicarbonate (phase B, pH=7.5). An isocratic elution program was performed, with phase A and phase B being mixed (90:10 proportion) at a flow rate of 0.2 ml/min. The injection volume was 5μL. The column oven was conditioned at +40°C. A TSQ Quantum triple quadrupole mass spectrometer (Thermo Fisher) using selected reaction monitoring (SRM) mode and an electrospray ionization source (ESI) in positive ion mode was utilized to obtain mass spectra at a voltage of 4000 V. The sheath gas pressure and auxillary gas pressure was maintained at 30L/min and 8L/min respectively. The capillary temperature was maintained at 380 °C throughout the run. The collision energy for the cytosine, 5-methylcytosine, 5-hmC and acyclovir were 21eV, 19eV, 19eV, and 21eV respectively. The precursor to product ion transition (Q1 to Q3) for quantitation (m/z) of cytosine, 5-methylcytosine, 5-hmC and acyclovir were programmed in the spectrometer at (112.2 to 95.3), (126.1 to 109.1), (142.0 to 81.3), and (226.2 to 152.1), respectively. The percentage of methylation was calculated using the following expression:

$$Methylation(\%)\;=\;Q_{5mCyt}/(Q_{5mCyt}+Q_{Cyt})\times 100\%$$ [1]

where *Q*_5*mCyt*_ is the molar quantity of 5-methylcytosine and *Q_Cyt_* is the molar quantity of cytosine determined in the DNA sample [38].

### Cell vitality assay

To evaluate the vitality of cells, the growth medium was disposed. Then wash the cells with PBS twice. 150μL 0.5mg/mL MTT solution was added to each well of 96 well plates. After incubation for 90min at 37°C with MTT, the supernatant in each well was removed. The precipitated formazan was solubilized with DMSO and quantified spectrophotometrically at 550nm.

### MMP assay

Following an incubation with the JC-1 at 37 °C/45 min, the culture medium was removed and plates were washed with PBS. Finally, fluorescence was measured in a Perkin Elmer LS-50B fluorescence microplate reader set at 525 nm (excitation) and 590 nm (emission).

### AKG sample preparation and HPLC condition

The cells were lysed by freeze-thaw cycles. The cell extractive was centrifuged for 10 min at 15000 rpm under 4°C. 100μL of the supernatant was stored at −70 °C until analysis.

The prepared samples are analyzed by a HPLC method according to the previous research with slight modifications [39]. The prepared samples were injected into a Agilent ZORBAX SB-Aq column (5.0μm, 150mm×2.0mm). The mobile phase consisted of water containing 25g/L Na_2_HPO_4_•12H_2_O. The flow rate of the mobile phase was 1 mL·min-1. The injection volume was 20μL. The column oven was conditioned at +40°C and UV detection is set to 210nm.

### Mechanism based kinetic progression model development

In this study (Figure 10B), the data for model development is collected in the above cell experiment. The mechanism based kinetic progression model is developed to investigate the dynamic process of cerebrovascular endothelial cell damage as well as the method for improving the cerebrovascular endothelial cell damage memory. This model has two major assumption. Firstly, the biomarkers introduced into this model fit the turnover equation which suggested that their production is depicted by zero-order process and their degradation is depicted by first-order process. Secondly, when the endothelial cells are exposed to a certain concentration of Aβ, the effect of Aβ is a constant. After the model is developed, simulations are performed for investigating the dynamic characters of cerebrovascular endothelial damage memory. The mechanism based kinetic progression model is described in the system which is composed of four linked turn over equations:

$$\begin{array}{l} \frac{dc_{Me-mtDNA}}{dt}=\;k_{in}^{Me-mDNA}-k_{out}^{Me-mtDNA}\\ \left(c_{Me-mtDNA}+\frac{E_{\max}^{AKG}c_{AKG}}{EC_{50}^{AKG}+c_{AKG}}-E_{A\beta }\right) \end{array}$$ [2]

$$\frac{dc_{AKG}}{dt}=k_{in}^{AKG}c_{MMP}-k_{out}^{AKG}c_{AKG}$$ [3]

$$\begin{array}{l} \frac{dc_{MMP}}{dt}=k_{in}^{MMP}-k_{out}^{MMP}(c_{MMP}\\ +\frac{E_{\max}^{Me-mtDNA}c_{Me-mtDNA}}{EC_{50}^{Me-mtDNA}+c_{Me-mtDNA}}) \end{array}$$ [4]

$$\begin{array}{l} \frac{dc_{MTT}}{dt}=k_{in}^{MTT}(1+\frac{E_{\max}^{MMP}c_{MMP}}{EC_{50}^{MMP}+c_{MMP}})\\ -k_{out}^{MTT}c_{MTT} \end{array}$$ [5]

The basal equation of mtDNA methylation (Eq. [2]) is depicted by a zero-order production rate ($k_{in}^{Me-mtDNA}$) and a first-order degradation rate ($k_{out}^{Me-mtDNA}$). In this system, Aβ may elevate the mtDNA methylation which is assumed to be constant at a certain concentration and described by parameter *E_Aβ_*. When the cell is incubated in growth medium without Aβ, the value of *E_Aβ_* is set to 1. As AKG is a vital cofactor of the demethylation enzymes ten-eleven-translocation (TET) enzymes, the effects of AKG on mtDNA demethylation is assumed to be described by a *E*_max_ model which contains two parameters $E_{\max}^{AKG}$ and $EC_{50}^{AKG}$ [40, 41]. The basal equation of AKG level (Eq. [3]) is depicted by a first-order production rate $\left(k_{in}^{AKG}\right)$ and a first-order degradation rate ($k_{out}^{AKG}$). The basal equation of MMP level (Eq. [4]) is depicted by a zero-order production rate ($k_{in}^{MMP}$) and a first-order degradation rate ($k_{out}^{MMP}$). The MMP collapse induced by mtDNA methylation is assumed to be described by a *E*_max_ model which contains two parameters $E_{\max}^{Me-mtDNA}$ and $E_{50}^{Me-mtDNA}$. The basal equation of cell vitality (Eq. [5]) is depicted by a constant of cell viability maintenance ($k_{in}^{MTT}$) and a reducing rate constant of cell viability ($k_{out}^{MTT}$). The cell vitality may be affect by MMP whose effect is assumed to be described by a *E*_max_ model including two parameters $E_{\max}^{MMP}$ and $EC_{50}^{MMP}$. The definition of parameters in the above model is shown in Table 2.

**Table 2:** The parameters of mechanism based kinetic progression mode for endothelial damage memory formation.

**Parameters**	**Definition**
**$k_{in}^{Me-mtDNA}$**	the methylation rate constant of mtDNA
**$k_{out}^{Me-mtDNA}$**	the demethylation rate constant of mtDNA
**$E_{\max}^{AKG}$**	The maximum effect of AKG on mtDNA demethylation
**$EC_{50}^{AKG}$**	a level of AKG at which half the maximum effect occurs
*E_Aβ_*	The effect factor of A β on mtDNA methylation
**$k_{in}^{AKG}$**	The production rate constant of AKG
**$k_{out}^{AKG}$**	The elimination rate constant of AKG
**$k_{in}^{MMP}$**	The generation rate constant of MMP
**$k_{out}^{MMP}$**	The collapse rate constant of MMP
**$E_{\max}^{Me-mtDNA}$**	The maximum effect of mtDNA hypermethylation on MMP collapse
**$EC_{50}^{Me-mtDNA}$**	a level of mtDNA methylation at which half the maximum effect occurs
**$k_{in}^{MTT}$**	The constant of cell viability maintenance
**$k_{out}^{MMP}$**	The maximum effect of MMP on cell viability maintenance
**$EC_{50}^{MMP}$**	a level of MMP at which half the maximum effect occurs
**$k_{out}^{MTT}$**	The reducing rate constant of cell viability

### Simulation

The simulation can provide insight into three issues. Firstly, the simulation can help find the time of cerebrovascular endothelial damage memory formation. For this scenario, Aβ is withdrawn at different time points and levels of mtDNA methylation, MMP and cell vitality are estimated to find the time at which the cell vitality may recover after Aβ is withdrawn. The formation of cerebrovascular endothelial damage memory is defined as that the cell vitality decreases more than 30% compared with control group and it can not recover after Aβ is withdrawn. Secondly, the influence factors for cerebrovascular endothelial damage memory formation are investigated by simulations. In this scenario, the effects of levels of mtDNA methylation and the TET cofactor AKG on cerebrovascular endothelial damage memory formation are investigated. When mtDNA methylation level or AKG level changes, the time for cerebrovascular endothelial damage memory formation is estimated. Thirdly, the methods for delaying the formation of endothelial damage memory are investigated by simulation. In this scenario, the endothelial improvement effect of mtDNA methylation inhibitor and AKG supplement are simulated. The improvement method proposed by simulation is validated in cell experiments.

### Simulation validation

**Figure 11:**
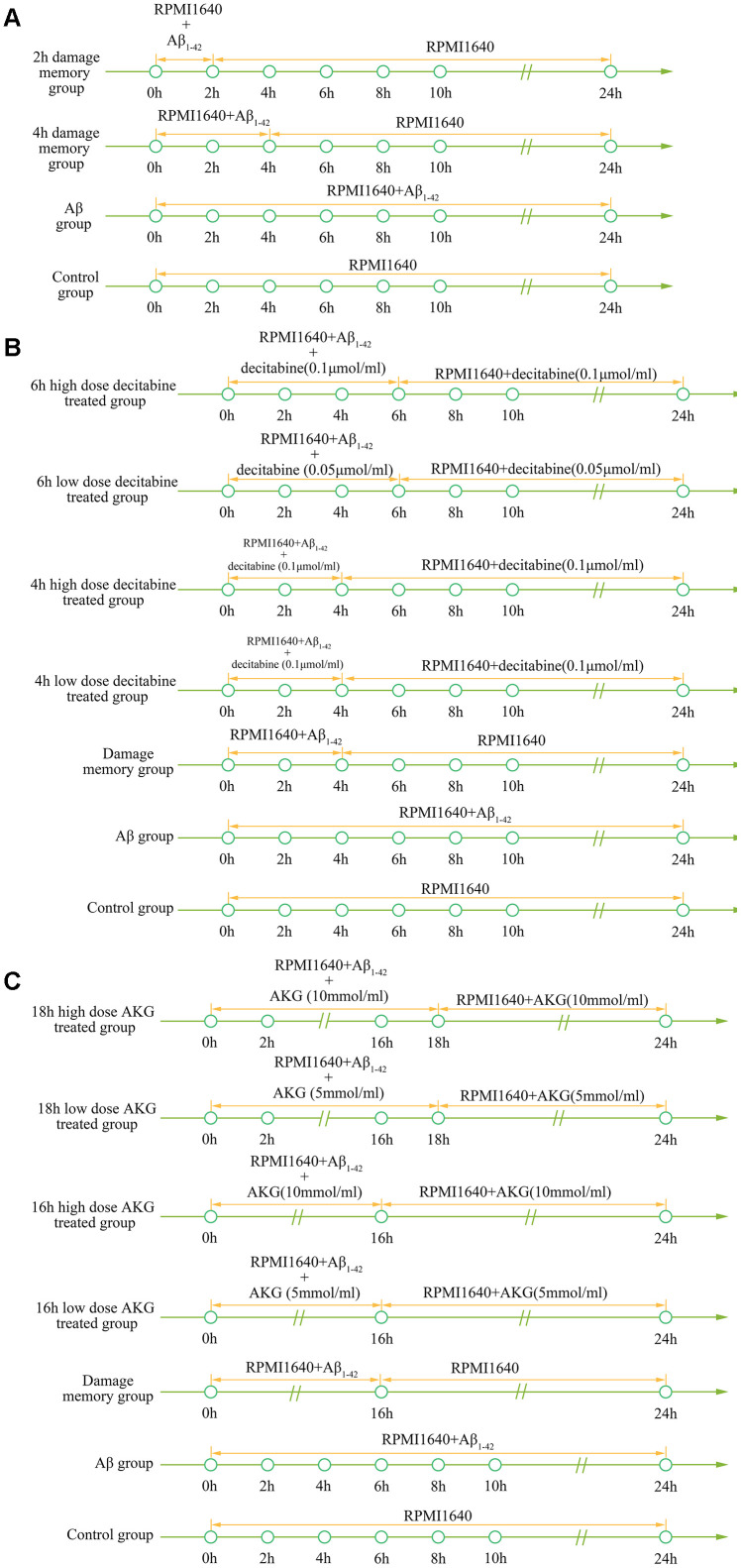
****(**A**) experimental protocol for determining time of cerebrovascular endothelial cell damage memory formation. (**B**) experimental protocol for evaluating the effect of decitabine on delaying the formation of cerebrovascular endothelial cell damage memory. (**C**) experimental protocol for evaluating the effect of AKG supplement on delaying the formation of cerebrovascular endothelial cell damage memory.

To validate the simulation based on the kinetic progression, three cell experiment are performed. The aim of the first cell experiment is to validate the baseline time of the formation of endothelial damage memory (Figure 11A). Validation of the baseline time could help us to compare the different endothelial function improvement methods. In the first experiment, the hCMEC/D3 cells were divided into four groups. In the first group (control group), the cells were incubated with complete RPMI 1640 for 24h. In the second group (2h memory group), the cells were incubated with culture medium containing *Aβ*_1–42_ for 2h and then withdraw *Aβ*_1–42_ for another 22h incubation. In the third group (4h memory group), the cells were incubated with culture medium containing *Aβ*_1–42_ for 4h and then withdraw *Aβ*_1–42_ for another 20h incubation. In the fourth group (Aβ group), the cells were incubated with culture medium containing *Aβ*_1–42_ for 24h. All the cell samples are collected for cell vitality measurement after 24h incubation.

The aim of the second experiment is to investigate the effects of DNA methylation inhibitor decitabine on delaying the formation of endothelial damage memory (Figure 11B). In this experiment, the cells were divided into seven groups. In the first group (control group), the cells were incubated with complete RPMI 1640 for 24h. In the second group (Aβ group), the cells were incubated with Aβ for 24h. In the third group (damage memory group), the protocol is same as the 4h memory group in the first experiment. In the fourth and fifth groups (4h high and low dose decitabine groups), the cells were incubated with culture medium containing *Aβ*_1–42_ for 4h and then withdraw *Aβ*_1–42_ for another 20h incubation meanwhile the cells were treated with 0.1 μmol/L and 0.05 μmol/L decitabine respectively during the entire incubation. In the sixth and seventh groups (6h high and low dose decitabine group), the cells were incubated with culture medium containing *Aβ*_1–42_ for 6h and then withdraw *Aβ*_1–42_ for another 18h incubation meanwhile the cells were treated with 0.1 μmol/L and 0.05 μmol/L decitabine respectively during the entire incubation.

The aim of the third experiment is to investigate the effects of AKG supplement on delaying the formation of endothelial damage memory (Figure 11C). In this experiment, the cells were divided into seven groups. The protocols of the first group (control group) and second (Aβ group) are same as those groups in the second experiment. In the third group (damage memory group), the cells were incubated with culture medium containing Aβ for 16h and then withdraw Aβ for another 8h incubation. In the fourth and fifth groups (16h high and low dose AKG groups), the cells were incubated with culture medium containing *Aβ*_1–42_ for 16h and then withdraw *Aβ*_1–42_ for another 8h incubation meanwhile the cells were treated with 10 mmol/L and 5 mmol/L AKG respectively during the entire incubation. In the sixth and seventh groups (18h high and low dose AKG groups), the cells were incubated with culture medium containing *Aβ*_1–42_ for 18h and then withdraw *Aβ*_1–42_ for another 6h incubation meanwhile the cells were treated with 10 mmol/L and 5 mmol/L AKG respectively during the entire incubation. All the cell samples were collected after 24h incubation for mtDNA methylation, AKG, MMP and cell vitality.
